# Identification of antimicrobial resistance genes in *Escherichia coli* through network diffusion

**DOI:** 10.1093/jac/dkaf404

**Published:** 2025-11-11

**Authors:** Anis Mansouri, Francesco Durazzi, Muhammad Ahmed Ihsan, Sholeem Griffin, Gerardo Manfreda, Vasilis P Valdramidis, Frédérique Pasquali, Daniel Remondini

**Affiliations:** Department of Physics and Astronomy, University of Bologna, Bologna, Italy; Department of Physics and Astronomy, University of Bologna, Bologna, Italy; Department of Food Sciences and Nutrition, Faculty of Health Sciences, University of Malta, Msida 2080, Malta; Department of Food Sciences and Nutrition, Faculty of Health Sciences, University of Malta, Msida 2080, Malta; Department of Agricultural and Food Sciences, University of Bologna, Bologna, Italy; Department of Food Sciences and Nutrition, Faculty of Health Sciences, University of Malta, Msida 2080, Malta; Department of Chemistry, Faculty of Sciences, National and Kapodistrian University of Athens, Athens, Greece; Department of Agricultural and Food Sciences, University of Bologna, Bologna, Italy; Department of Physics and Astronomy, University of Bologna, Bologna, Italy

## Abstract

**Objectives:**

Antimicrobial resistance (AMR) is an escalating global health concern, driven by multifactorial biological processes not yet fully understood. This study employed network diffusion analysis to dissect the molecular mechanisms driving AMR in *Escherichia coli*, aiming to identify novel potential drug targets for therapeutic development.

**Methods:**

A systems biology approach was used to identify genes and biological pathways associated with AMR, by mapping known AMR-related genes from the Comprehensive Antibiotic Resistance Database (CARD) and PointFinder database into the *E. coli* protein interactome. Through a network diffusion algorithm, several network modules were identified, i.e. genes and pathways, in part already known to be involved in AMR mechanisms. We selected gene candidates for performing an *in vitro* susceptibility validation test, consisting of 13 knockout mutants against nine different antibiotics.

**Results:**

Compared with the WT *E. coli* BW25113, the AMR of some mutants showed significant shifts of biological relevance: Δ*uhpB* (S/I) and Δ*mdaB* (S/R) against ampicillin, Δ*rpmG* (I/S) and Δ*rplA* (I/S) against ciprofloxacin, and Δ*rplA* (I/S) against streptomycin (S, susceptible; I, intermediate; R, resistant). In other cases, only a significant change in inhibition disc diameter was observed, probably deserving further studies.

**Conclusions:**

Network diffusion is an effective tool to infer relevant biological insights related to AMR from microbial biological networks. Our results contribute to a better understanding and characterization of AMR in *E. coli.* Furthermore, the *in vitro* validated genes could be considered as new putative drug targets.

## Introduction

The continual increase in antimicrobial resistance (AMR) concerns public health. Of 4.95 million deaths caused by microbial infections, bacterial AMR was estimated to be directly responsible for 1.27 million deaths globally in 2019.^1^ The misuse of antimicrobial agents can cause bacteria and other pathogens to adapt by developing protective mechanisms such as mutations, metabolic adaptations and secretion of anti-antimicrobial molecules, leading to severe illness, disability and death.^2–4^ The emergence of MDR or pan drug-resistant bacteria suggests the need to focus research on new antimicrobials. In the past, the approach to drug discovery was essentially based on biological information linked explicitly to the target and mechanism of action of the molecule. With the progress in omics and computational sciences, the scientific community is increasingly aware that genes and proteins do not act as standalone molecules but interact on multiple hierarchical levels as complex networks.^5–7^ Protein–protein interaction (PPI) networks aim to grasp this complex pattern of interactions by modelling individual proteins as nodes and their relationships as undirected edges. PPI information can be retrieved from various resources based on known and/or computationally predicted interactions. In online resources such as STRING,^8^ GeneMANIA,^9^ FunCoup^10^ and ConsensusPathDB,^11^ experimental data are integrated with interaction prediction algorithms, thus aiming for high comprehensiveness and coverage.

Meaningful biological insights can be extracted through different network-based analyses. Network diffusion (ND) is used in many contexts, such as gene prioritization, function prediction, survival prediction and disease subtyping.^12^ ND relies on the propagation of information (‘signal’) from already characterized source nodes (‘seed’ genes) through the network. Based on the guilt-by-association principle,^13^ nodes that are adjacent to the source nodes will accumulate more signal after diffusion, and thus they will be more likely related to the phenotypic trait of interest.^14^ ND has been used for different purposes such as the prediction of functional associations of unannotated gene sets,^15^ the prediction of protein functions,^16^ the identification of cancer gene mutations,^17^ or of new COVID-19 drug targets.^18^ Several studies have highlighted the relevance of gene networks in identifying and elucidating AMR mechanisms in different microbial pathogens.^19^

*Escherichia coli* is a widely used model organism. It is easy to grow, fast in replication and amenable to genetic manipulation, making it the most studied organism on the planet.^20,21^ For food safety, *E. coli* is under mandatory surveillance as a bacterial indicator of AMR circulating in livestock, food and humans.^22^ The present work integrates the *E. coli* STRING protein-protein network with curated AMR gene datasets from the Comprehensive Antibiotic Resistance Database (CARD)^23^ and PointFinder database,^24^ to perform ND aimed at identifying novel AMR-associated genes. Subsequently, we conducted experimental validation of the top-ranking genes not previously associated with AMR or tested under narrower antibiotic conditions. The results contribute substantially to deepening the knowledge of existing AMR mechanisms and provide the foundation for the discovery of novel antimicrobial targets against *E. coli*.

## Methods and materials

### Protein–protein interaction networks

Eleven *E. coli* strain PPIs were available on the STRING database v11.5. Only two, *E. coli* CFT073 and *E. coli* K12 MG1655, were core species for which a fully sequenced genome and experimental data were available.^25^ We used the PPI network of *E. coli* K12 MG1655 as it is a widely studied laboratory strain. The PPI undirected interactions were collected based on the ‘b number’ *E. coli* gene identifiers for each node. A total of 4053 nodes and 33 656 edges were retained, considering only relationships between proteins characterized by a high confidence score (≥0.7) as provided by the STRING website.

### Mapping known AMR genes on E. coli K-12 MG1655 interactome

CARD (v3.2.7) and PointFinder (v.4.1.0) were selected as comprehensive databases of AMR-related genes.^23,24^ We collected a list of 34 AMR-related genes from the two databases that could be mapped onto the chosen PPI (Table 1): 32 genes from CARD, and 2 from PointFinder. These genes were used as ‘seed genes’ for the ND procedure (see Methods - Network-Based Analysis).

**Table 1.** CARD and PointFinder genes mapped to *E. coli* K-12 MG1655 interactome

b#	Gene symbol^a^	CARD	PointFinder
B0463	*acrA*	X	
B0464	*acrR*	X	
B0543	*emrE*	X	
B0578	*nfsB*	X	
B0842	*mdfA*	X	
B0851	*nfsA*	X	
B0929	*ompF*	X	
B1093	*fabG*	X	
B1288	*fabI*	X	
B1530	*marR*	X	
B1782	*mipA*	X	
B2231	*gyrA*	X	X
B2240	*glpT*	X	
B2416	*ptsI*	X	
B3019	*parC*	X	X
B3030	*parE*	X	X
B3177	*folP*	X	X
B3189	*murA*	X	
B3666	*uhpT*	X	
B3669	*uhpA*	X	
B3699	*gyrB*	X	X
B3806	*cyaA*	X	
B3912	*cpxR*	X	
B3987	*rpoB*	X	X
B4036	*lamB*	X	
B4062	*soxS*	X	
B4063	*soxR*	X	
B4150	*ampC*	X	X
B4396	*rob*	X	
B4113	*basR/pmrA*	X	X
B4112	*basS/pmrB*	X	X
B0084	*ftsI(PBP3)*	X	
B3339	*tufA/EFTu*	X	
b3980	*tufB/EFTu*	X	

^a^‘Gene_symbol’ reports the gene symbols and their synonyms, if any.

The seed gene list comprises the genes annotated with the prefix ‘Ecol_’ according to CARD annotation. Genes related to AMR in *E. coli* without this prefix are used as a literature-based validation gene set to assess how well the diffusion approach can recover genes already known to be involved in AMR.

### Network-based analysis

An ND algorithm,^26^ from the R package diffuStats v1.24, was employed by taking as input the initial 34 AMR genes list and the *E. coli* K-12 MG1655 PPI network.^27^ The ND process simulates the spread of information within a network, analogous to fluid diffusing along a pipe system. The fluid starts from ‘seed’ sources (our AMR-related seed genes) and it spreads to other genes. The amount of information (i.e. fluid) retained by each node (i.e. gene) after the diffusion process reaches a stationary state, which we quantify with the diffusion score S*_x_* measuring the involvement of each gene in the AMR process.^26^ Since biological systems are known to have a highly modular structure, this approach helps to identify the gene modules associated to a specific feature (in our case putative genes related to AMR). To quantify the statistical significance of the scores, a permutation procedure was applied by running 1000 diffusions on the network with 34 randomly assigned seed genes at each iteration. A *P* value for each S_x_ score was then computed (*P* value = proportion of random scores  ≥ S_x_ score). Genes having an S_x_ score with a *P* value ≤0.01 were selected as top-ranking AMR-related genes. Finally, an induced subnetwork comprising the top-ranking genes was extracted from the whole *E. coli* K-12 MG1655 PPI, for which a community detection algorithm^28^ was used to identify gene clusters that could be associated with specific biological functions. All network analyses were performed via the igraph R package.^29^

### Pathway enrichment analysis

KEGG pathway annotation was used for the identification of overrepresented pathways.^30^ The genes selected through the S_x_ score were used to perform an overrepresentation analysis via the R package clusterProfiler.^31^ The *P* values of the enrichment analysis were corrected for multiple testing by using the Benjamini–Hochberg *post hoc* method.^32^ A false discovery rate (FDR) ≤0.05 was chosen for significant pathway overrepresentation.

### In vitro validation through antibiotic susceptibility testing

Starting from the gene list obtained through ND, we identified a subset of genes to be experimentally validated, as available in the Keio collection.^33^ We filtered out genes based on the following criteria: (i) seed genes; and (ii) essential genes (i.e. genes for which the knockout is fatal for the bacterial cell). From this filtered list, we sampled a subset of genes for experimental validation, considering all the remaining ones as equally suitable (Table S1, available as Supplementary data at *JAC* Online). The antibiotic susceptibility testing (AST) in *E. coli* isolates was conducted using the Kirby–Bauer disc diffusion method following the protocols outlined by the CLSI.^34^ A total of nine antibiotics were assessed: ampicillin (10 μg), chloramphenicol (30 μg), ciprofloxacin (5 μg), fosfomycin (200 μg), penicillin G (10 units), polymyxin B (300 units), spectinomycin (10 μg), streptomycin (10 μg) and tetracycline (30 μg). Initially, the *E. coli* isolates were cultured in nutrient broth (Himedia, India) and then incubated at a temperature of 35 ± 2°C for 18–24 h. The bacterial suspension was subsequently standardized to a 0.5 McFarland turbidity standard, resulting in a concentration of around 10^8^ cfu/mL. Employing cotton swabs, the bacterial suspension was uniformly distributed on Mueller–Hinton agar plates (HiMedia Laboratories, Mumbai) and allowed to air dry for 15 minutes. The antibiotic discs were positioned on the agar surface with a minimum distance of 30 mm between each disc. Subsequently, the plates were inverted and aerobically incubated at a temperature of 35 ± 2°C for 16–18 h. The zones of inhibition were quantified using an automated colony counter (Interscience Scan500) and interpreted in line with the recommendations provided by the CLSI.^35^ To ensure quality control, the *E. coli* ATCC 25922 strain was used.

For each tested antibiotic, the average inhibition diameters of the mutants were compared with the WT using an unpaired Student's *t* test. The *P* values were adjusted for multiple hypothesis testing using the Benjamini–Hochberg *post hoc* correction for multiple tests.^32^ The statistical analysis was carried out using R.^36^

## Results

### Network-based and pathway analyses

**Figure 1.**
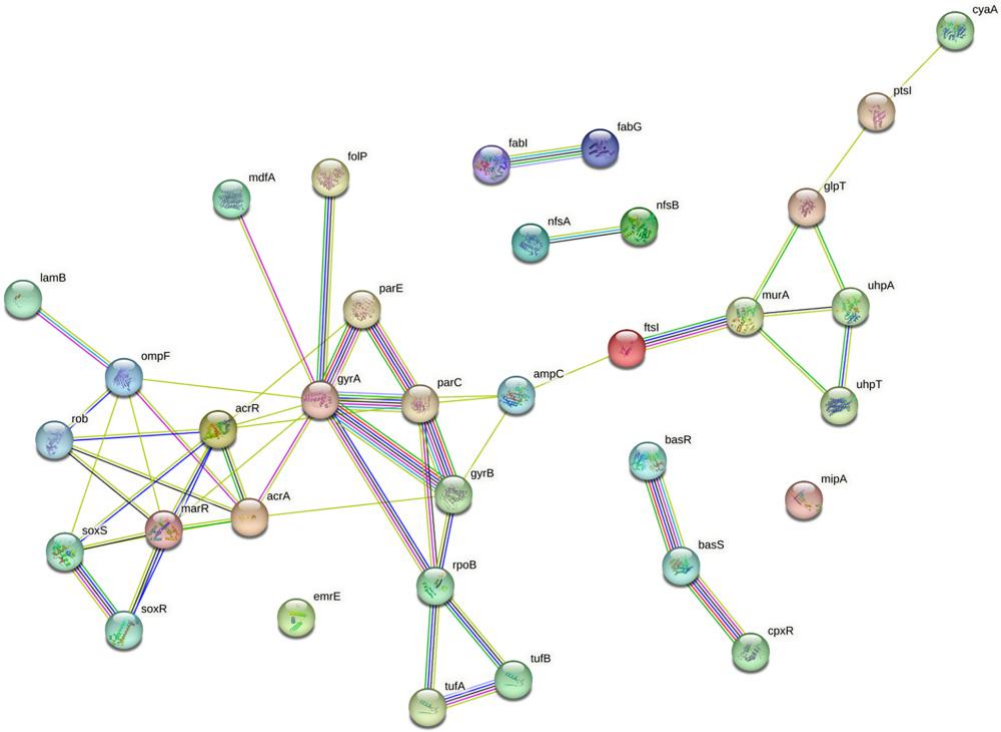
Subnetwork of the 34 known antimicrobial genes (seed genes) with the links found in the *E. coli* PPI STRING interactome. The interaction diagram was generated by https://string.db.org.

The 34 AMR-related seed genes (Figure 1) belong to six KEGG pathways: β-lactam resistance, cationic antimicrobial peptide (CAMP) resistance, two-component system, fatty acid biosynthesis, fatty acid metabolism and biotin metabolism. These results reflect the complexity of the antimicrobial mechanisms that involve more than one biological pathway.^37,38^

**Figure 2.**
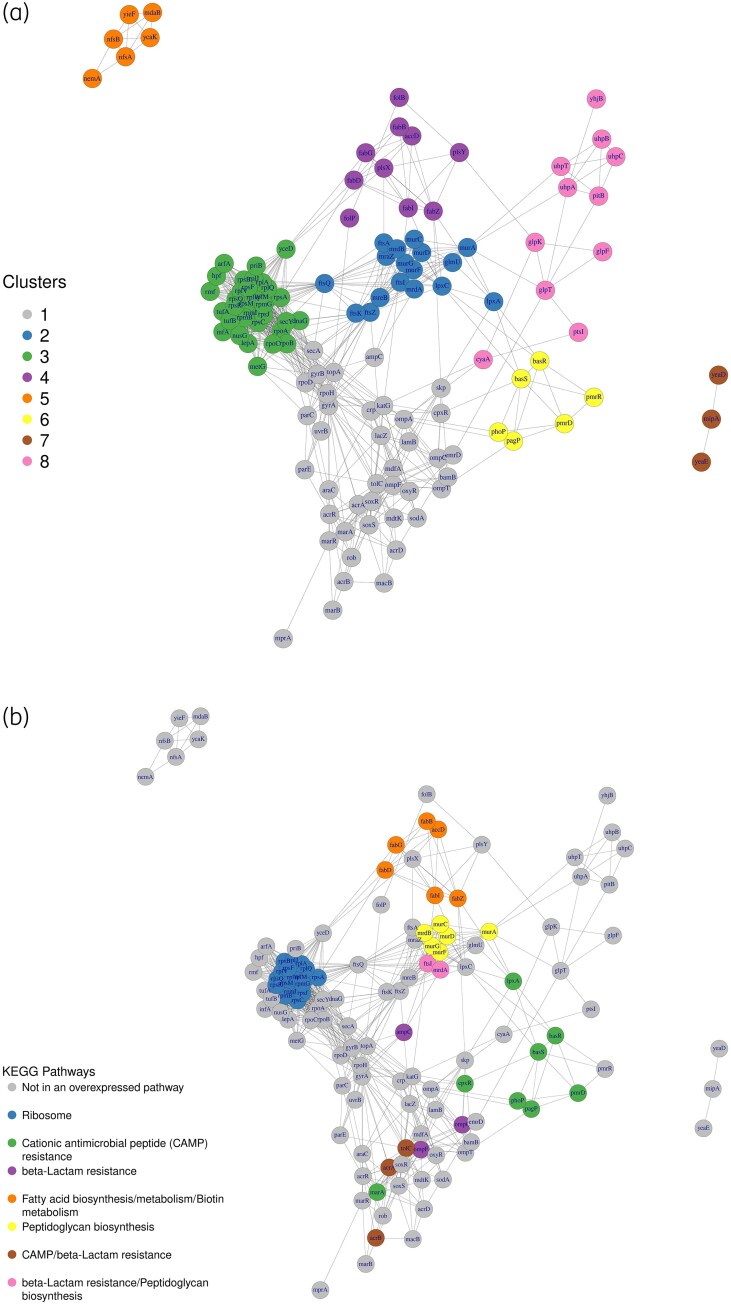
(a) The induced subnetwork of *E. coli* K-12 MG1655 interactome with 127 genes coloured by membership to one of the eight clusters identified by the community detection (CD) algorithm. (b) Plot of the same network with genes coloured by KEGG pathway annotation to highlight the overlap between the clusters defined by the CD algorithm and KEGG curated pathways. This allows assessment of the capacity of the CD algorithm to identify clusters having biological meaningfulness. The gene *emrE* is not shown as it has no links with the genes represented in this figure. Nevertheless, *emrE* interacts with genes in the remaining part of the PPI.

The ND generated a list of 127 genes associated with AMR: 34 are the original seed genes, whereas the remaining 93 constitute the novel result (list in Table S1). We considered the PPI subnetwork induced by the 127 gene list, consisting of one large, connected component of 117 nodes, two small components of 6 and 3 nodes and 1 isolated node. In Table 2 we show the results of the pathway enrichment analysis on the 117-gene component. Seven enriched pathways were identified: ribosome, CAMP, β-lactam resistance, fatty acid biosynthesis, peptidoglycan biosynthesis, fatty acid metabolism and biotin metabolism. These pathways overlap mostly with those related to the 34 seed genes, suggesting consistency with known AMR pathways in *E. coli*. In Figure 2 we show the PPI subnetwork of the 127 genes detected with ND; in Figure 2a the colouring depicts network communities identified via community detection whereas in Figure 2b the colouring depicts the KEGG pathway of each gene. The pathways partially overlap with the communities: the ribosome pathway overlaps with cluster 3, the CAMP pathway with cluster 6, β-lactam resistance with cluster 1, fatty acid biosynthesis/metabolism and biotin metabolism, which share many genes, overlap with cluster 4, and peptidoglycan biosynthesis overlaps with cluster 2.

**Table 2.** Results of the pathway enrichment analysis^a^

Pathway	FDR	No. of genes in the network	Gene symbols	Total no. of genes in the pathway	Seed genes in the pathway
Ribosome	4.43 × 10^−7^	17	*rpsB*, *rpsA*, *rpmI*, *rplM*, *rplQ*, *rpsM*, *rpsQ*, *rpsC*, *rplV*, *rplW*, *rpsJ*, *rpmG*, *rpmB*, *rplA*, *rplJ*, *rpsF*, *rpsR*	78	—
Cationic antimicrobial peptide (CAMP) resistance	2.3 × 10^−6^	11	*lpxA*, *acrB*, *acrA*, *pagP*, *phoP*, *marA*, *pmrD*, *tolC*, *cpxR*, *basS*, *basR*	36	*acrA*, *cpxR*, *basS*, *basR*
β-Lactam resistance	2.3 × 10^−6^	8	*ftsI*, *acrB*, *acrA*, *mrdA*, *ompF*, *ompC*, *tolC*, *ampC*	17	*ftsI*, *acrA*, *ompF*, *ampC*
Fatty acid biosynthesis	8 × 10^−5^	6	*fabZ*, *fabD*, *fabG*, *fabI*, *accD*, *fabB*	13	*fabG*, *fabI*
Peptidoglycan biosynthesis	3.7 × 10^−4^	7	*ftsI*, *murF*, *murD*, *murG*, *murC*, *mrdA*, *murA*	24	*ftsI*, *murA*
Fatty acid metabolism	1.3 × 10^−3^	6	*fabZ*, *fabD*, *fabG*, *fabI*, *accD*, *fabB*	21	*fabG*, *fabI*
Biotin metabolism	1.4 × 10^−2^	4	*fabZ*, *fabG*, *fabI*, *fabB*	14	*fabG*, *fabI*

^a^For each of the overrepresented pathways we show the false discovery rate (FDR) as a significance metric, the number and symbols of the genes in the network mapped to the pathway, the number of total genes mapped to the pathway according to the KEGG, and the seed genes mapped to the pathway, if any.

The ribosome pathway was the most significant and largest enriched pathway, with 17S, 30S and 50S ribosomal subunit proteins responsible for decoding mRNAs and control of translation fidelity, and the catalysis of protein synthesis, respectively.^39^ Interestingly, none of them was a seed gene, but we found four seed genes (*tufA*, *tufB*, *rpoB* and *gyrA*) in their first-order neighbourhood (see Figure S1), which represent important targets of many antimicrobial therapeutic strategies, such as miscoding using streptomycin and paromomycin,^40^ minimization of ribosomal mobility,^41^ and blockage of the protein exit tunnel.^42^ The CAMP resistance pathway was the second largest enriched pathway (11 genes). The cationic peptides (CPs) are antimicrobial components naturally expressed by animals, plants and even bacteria.^43^ Through electrostatic interactions, they bind the outer layer of the bacterial cytoplasmic membrane and induce lysis of the targeted microbial cell.^44^ Bacteria can acquire resistance to CPs through different mechanisms, such as the modification of the cell surface structure and its net charge in Gram-negative bacteria to alter CP binding.^43^ Another CAMP resistance mechanism previously observed in *E. coli* and *Staphylococcus aureus* relies on the trapping and proteolytic degradation of CPs by production of metalloproteinases.^45^ Moreover, it has been shown that *E. coli* and other bacteria have developed another strategy to survive in CP-rich environments. This strategy consists in the release of negatively charged capsular polysaccharides to neutralize and titrate CPs by means of electrostatic interactions.^46^ β-Lactams are antibacterials designed to inhibit cell wall synthesis: β-lactam resistance (third overrepresented pathway) is one of the earliest characterized and most successful AMR strategies.^47,48^ Fatty acid, peptidoglycan and biotin biosynthesis/metabolism pathways contribute to AMR resistance through global cell adaptation such as metabolic adaptations and cell envelope homeostasis, and are the object of research to identify novel antimicrobial targets.^49^  *E. coli* can acquire resistance to antibacterial agents, such as triclosan, that inhibit its lipid synthesis by altering its target, the enoyl-[acyl-carrier-protein] reductase *fabI*. A missense mutation in *fabI* hinders triclosan activity by reducing the binding affinity of the complex FabI-triclosan.^50^ Survival strategies relying on membrane homeostasis are found in many pathogens to increase their fitness in the presence of environmental stressors such as antibiotics.^51^ It has been observed that increased tPMP (thrombin-induced platelet microbicidal protein) resistance in *S. aureus* is due to a higher cell membrane fluidity caused by a preponderance of longer chain, unsaturated fatty acids.^52^

Peptidoglycan sacculus (PS) is an elastic, net-like polymer that surrounds the cytoplasmic membrane in most bacteria. It contributes to the preservation of cell integrity during growth and division and the protection of the bacterium against environmental challenges such as osmotic stress.^53^ PS structure is disrupted by β-lactams that target the PBPs responsible for the synthesis of the 4→3 peptidoglycan cross-linking. To cope with the effects of β-lactams, *E coli* expresses the L,d-transpeptidase YcbB, which catalyses an unusual 3→3 peptidoglycan cross-linking to maintain cell wall integrity.^54^ We remark that our analysis did not identify novel pathways, but it was able to retrieve pathways known to be AMR-associated thus providing an indirect validation of the goodness of our approach.

### Literature validation of the prioritized AMR genes

In Table 3 we list the genes with the highest diffusive S_x_ score in each overrepresented pathway, excluding the seed genes. Except for *rpmB*, all these genes are already known to be associated with AMR in many microorganisms, including *E. coli*.

**Table 3.** List of top-ranking genes for each pathway (excluding seed genes) and their reference in literature as AMR genes

Gene	S_x_	Annotated in overrepresented pathways	Source
*uhpB* *uhpC*	0.26 0.25	No	^55^
*rpmB* ^a^ *rpsj* *rpmG*	0.027 0.026 0.026	Ribosome	No source,^56^ CARD
*marA* *pmrD*	0.1 0.05	Cationic antimicrobial peptide (CAMP) resistance	^57^, CARD
*ompC*	0.04	β-Lactam resistance	^58,59^
*rabD* *rabB* *fabZ*	0.05 0.04 0.04	Fatty acid biosynthesis/metabolism/biotin metabolism	^60–62^
*murC*	0.04	Peptidoglycan biosynthesis	^63^
*tolC* *acrB*	0.09 0.07	Cationic antimicrobial peptide (CAMP) resistance and β-lactam resistance	CARD
*mrdA(PBP2)*	0.04	β-Lactam resistance and peptidoglycan biosynthesis	^64^

^a^ *rpmB* is an essential gene and has the highest diffusion score among the ribosomal genes; nevertheless no source confirming its implication in AMR was found.

### In vitro antibiotic susceptibility of mutants

In order to phenotypically investigate their role in AMR, 13 newly identified genes were selected for performing experiments with selected knockout among the 52 genes that satisfied the criteria described in the Methods - In Vitro Validation Through Antibiotic Susceptibility Testing section (see also Table S1): *uhpB* (JW3643-KC), *mdaB* (JW2996-KC), *yieF* (JW3691-KC), *pitB* (JW2955-KC), *rplA* (JW3947-KC), *uvrB* (JW0762-KC), *rpmG* (JW3611-KC), *rpsF* (JW4158-KC), *nemA* (JW1642-KC), *ompC* (JW2203-KC), *ompT* (JW0554-KC), *yeaD* (JW1769-KC) and *yeaE* (JW1770-KC). UhpB is a sensor of the HK protein that controls the production of the sugar phosphate transporter UhpT.^65^ The G469R mutation in the *uhpB* gene is associated with fosfomycin resistance.^66^ The susceptibility of the knockout mutant to other antimicrobials with mechanisms of action similar to that of fosfomycin has not yet been investigated. MdaB is an NADPH oxidoreductase that protects cells against quinonoid compounds.^66,67^ This protein is known to confer resistance to the antibiotics DMP 840, adriamycin and etoposide.^67^ YieF is a chromium reductase involved in bacterial tolerance to this heavy metal.^68^ PitB is involved in phosphate transport.^69^ In the cell, orthophosphates are suggested to link heavy metals, and metal phosphates are transported out of the cell by PitB thus contributing to heavy metal resistance.^69^ RplA is a ribosomal protein; knockout of the corresponding gene has been associated with zinc resistance.^70^ Co-resistance to antimicrobials and heavy metals is described as synergistic with the potential for antimicrobial resistance.^71^ Heavy metals promote the spread of AMR genes and bacteria in the environment.^66^ UvrB is involved in the SOS response associated with DNA biosynthesis and repair.^72^ Another protein involved in DNA repair is RpmG, which is associated with resistance to mitomycin C, a natural antimicrobial synthesized by *Streptomyces caespitosis* and associated with DNA damage.^73^ The *rpsF* gene encodes an S6 ribosomal protein.^74^ Mutations in ribosomal proteins have been described as associated with erythromycin, spectinomycin and streptomycin resistance in *E. coli.*^75^ The *yeaD* gene encodes the D-hexose-6-phosphate epimerase-like protein, which is involved in galactose metabolism.^76^ Bacterial epimerases are involved in the formation of complex carbohydrate polymers that are constituents of cell walls and cell membranes.^77^ The *nemA* gene encodes *N*-ethylmaleimide reductase in *E. coli*.^78^ The presence of the gene is associated with higher resistance of *E. coli* to acid hydrolysate of sugarcane bagasse.^79^ The correlation between acid tolerance and AMR has been previously described.^80^ OmpT is a protease located on the outer membrane and participates in the adhesion of *E. coli* O157:H7 to human epithelium.^81^

Table 4 shows the susceptibility test results of the selected mutants with respect to the WT. Knockout mutants of *uhpB*, *mdaB*, *rpmG* and *rplA* showed a statistically significant variation of their antimicrobial susceptibility compared with the WT, supporting their functional contribution to AMR mechanisms. Specifically, the *mdaB* mutants demonstrated resistance to ampicillin, whereas *rpmG* and *rplA* mutants showed a shifted AST against ciprofloxacin, with *rplA* also displaying resistance to streptomycin. Some knockout mutants did not display a change in AMR susceptibility class relative to the WT, even if their inhibition zone diameter was significantly different than the WT. This might indicate either non-involvement in AMR, or potential synergistic effects with other genes yet to be investigated.

**Table 4.** Susceptibility tests of the selected mutants compared with the WT, according to CLSI standards

	Streptomycin	Ciprofloxacin	Ampicillin	Tetracycline	Chloramphenicol	Fosfomycin
WT	I	I	S	S	S	S
Δ*uhpB*	I	I	I^a^	S	S^a^	S^a^
Δ*mdaB*	I	I^a^	R^a^	S	S	S
Δ*rpmG*	I	S^a^	S^a^	S^a^	S	S^a^
Δ*rplA*	S^a^	S^a^	S^a^	S^a^	S^a^	S^a^

S, susceptible; I, intermediate; R, resistant.

^a^The inhibition zone diameter of the mutant is significantly different than the WT (*P* value ≤0.01). Some mutants did not shift their AMR susceptibility class even if their inhibition zone diameter was significantly different than the WT.

## Discussion

In the present study, several genes and biological pathways associated with AMR were identified (see Tables 2, 3 and 4). The ribosome pathway is already observed as enriched in other microorganisms, namely *S. aureus, Clostridioides difficile, Helicobacter pylori* and *Campylobacter jejuni.*^82–84^ CAMP, β-lactam resistance and peptidoglycan biosynthesis pathways were previously reported as relevant pathways in AMR mechanisms in *S. aureus*, *Salmonella* Typhi and *E. coli* O157:H7.^85^ Fatty acid biosynthesis was reported as significantly enriched in *E. coli* O157:H7.^86^ Not surprisingly, genes related to efflux pumps (i.e. *acrAB*, *tolC*), were identified in this study as in previous ones.^87^ By actively extruding antibiotics from the cell, multidrug efflux pumps have been under the lens of researchers for the last two decades as potential targets for novel drugs able to revert phenotypes resistant to several antibiotics.^88^ New genes not previously identified by systems biology approaches are *uhpB*, *uhpC* and *mdaB*. UhpB is a sensor histidine kinase of a two-component system (TCS). TCSs have been previously highlighted as primary pathways by which bacteria adapt to environmental stresses such as antibiotics. Knockout mutants of TCS genes enriched in the present study, namely *phoP*, *cpxR* and *basR*, have shown significant shifts in their antimicrobial susceptibility, reinforcing their role in AMR mechanisms.^87^

Differently from previous findings, *uhpB* mutants were not fosfomycin resistant. The gene *uhpB* is an activator of the expression of *uhpT*, which encodes a phosphate-inducible transporter responsible for the uptake of small molecules.^55^ Results in the present study suggest the potential involvement of *uhpT* in ampicillin uptake. Regarding MdaB, further studies are needed to elucidate if this enzyme can inactivate ampicillin similarly to its detoxification role against quinones. For ciprofloxacin, the knockout mutants *rpmG* and *rplA* were susceptible to this antimicrobial whereas the WT expressed an intermediate phenotype. RpmG is already known to be involved in DNA repair; its role in the ciprofloxacin-susceptible phenotype might be associated with the repair of DNA damage due to the inhibition of DNA synthesis by this antibiotic. RplA is a ribosomal protein with no apparent connections with ciprofloxacin’s mode of action. *rplA* mutants were also susceptible to streptomycin whereas the WT was intermediate and carried the *strA* gene. Although without a significant shift in the antimicrobial susceptibility, *rplA* mutants showed a significantly higher zone diameter than the WT (*P* < 0.01, see Table 4) for all six antibiotics, suggesting that *rplA* might be involved in the mechanism of intrinsic resistance, since its deletion is responsible for higher sensitivity compared with the WT. Additional studies are needed to confirm and further investigate the potential role of *rplA* in intrinsic MDR or reduced susceptibility.

The diffusion process proved instrumental in revealing the association between AMR and ribosomes (Table 2), which serve as critical targets for various antimicrobial therapeutic strategies, even though the network was not seeded with any ribosomal gene. This suggests that the outcomes of the ND gene prioritization are not limited by the initial seed genes. ND is an intuitive and easy-to-implement algorithm as it requires only two inputs: the initial seed genes playing the role of information source, and a network where this information is propagated. Therefore, ND can be applied to study any kind of organism if these inputs are available. Hence, the quality of ND’s outputs depends on: (i) the availability of well-characterized seed genes in literature or databases, and (ii) the availability of good quality networks with well-annotated and validated interactions. Due to their complexity, curated biological networks are available for only a limited number of organisms: the STRING database v11.5 contains annotations for about 14 000 organisms. This is a limiting factor making ND unsuitable for organisms lacking well-characterized gene/protein interactions. Another possible limiting factor is an incomplete knowledge of seed genes, which could introduce biases in the method by completely missing exploration of PPI regions for which seed genes are not available. Moreover, this approach cannot deal with mobile genetic elements, such as plasmids, if their interactions with the core genome network mapped into the PPI are not known. We also remark that we might have missed potential AMR-related genes with our selection criteria for *in vitro* validation. Thus extended experimental testing could be the object of further studies, also considering possible synergies of multiple genes.

## Supplementary Material

dkaf404_Supplementary_Data

## Supplementary data

Figure S1 and Table S1 are available as Supplementary data at *JAC* Online.
