# Supplementary material for: Identification of antimicrobial resistance genes in Escherichia coli through network diffusion
Source: J Antimicrob Chemother. 2025 Nov 11;81(1):dkaf404. doi: 10.1093/jac/dkaf404 (PMC12802890; doi:10.1093/jac/dkaf404)
Supplement: dkaf404_Supplementary_Data [file dkaf404_supplementary_data.zip › Ecoli AMR Revised highlight SUPPLEMENTARY 26set25.docx]

Identification of antimicrobial resistance genes in *Escherichia coli* through network diffusion – Supplementary Material

Anis MANSOURI^1^*, Francesco DURAZZI^1^*, Muhammad Ahmed IHSAN^2^*, Sholeem GRIFFIN^2^, Gerardo MANFREDA^4^, Vasilis P. VALDRAMIDIS^2,3^, Frédérique PASQUALI^4^, Daniel REMONDINI^1+^

1 Department of Physics and Astronomy, University of Bologna, Bologna, Italy.

2 Department of Food Sciences and Nutrition, Faculty of Health Sciences, University of Malta, 2080 Msida, Malta.

3 Department of Chemistry, Faculty of Sciences, National and Kapodistrian University of Athens, Athens, Greece.

4 Department of Agricultural and Food Sciences, University of Bologna, Bologna, Italy.

* Equal contribution

^+^ Corresponding author: daniel.remondini@unibo.it


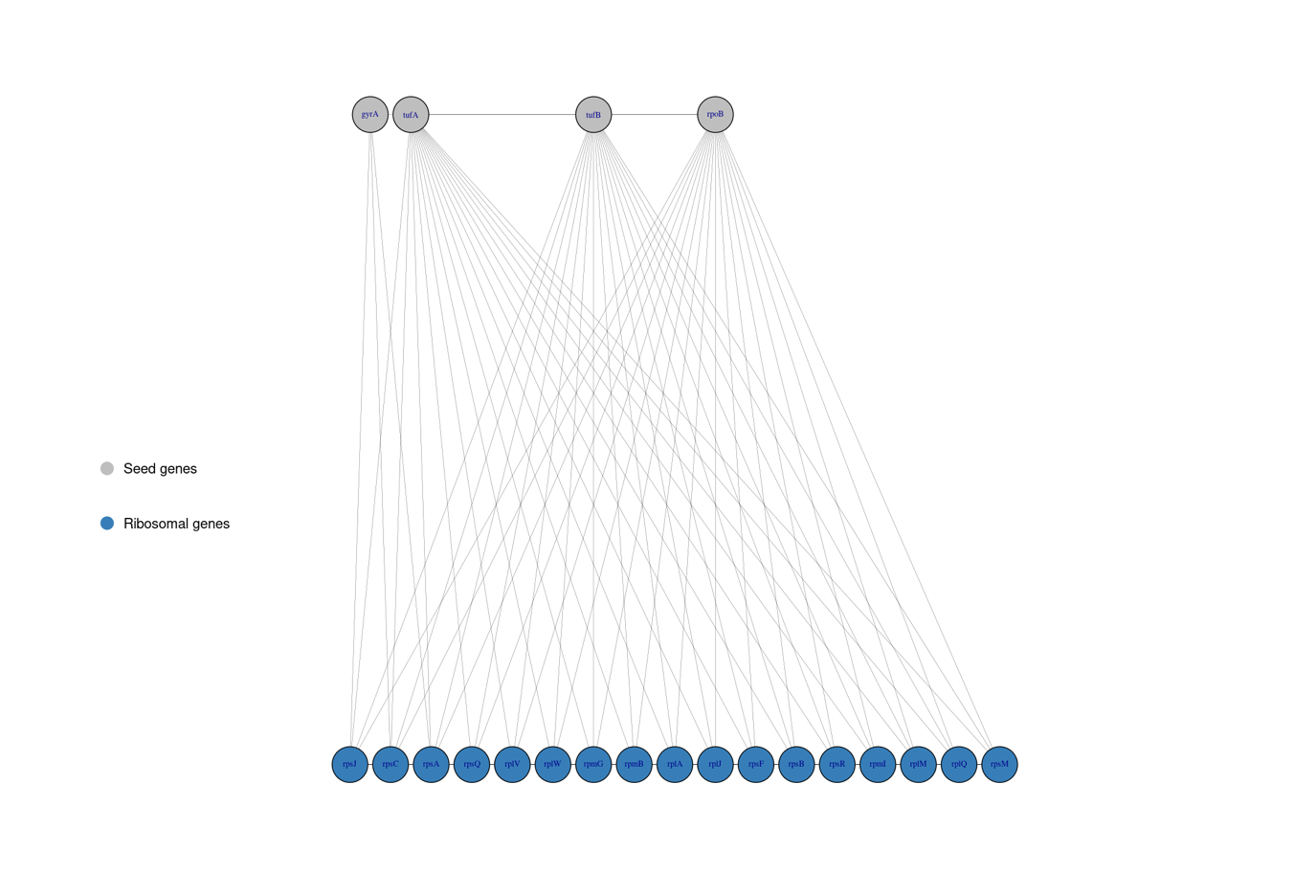


***Figure S1:*** *network representation of the ribosomal genes’ first-order neighborhood highlighting links with four seed genes tufA, tufB, rpoB and gyrA.*
